# Expression of an immunocomplex consisting of Fc fragment fused with a consensus dengue envelope domain III in *Saccharomyces cerevisiae*

**DOI:** 10.1007/s10529-021-03161-7

**Published:** 2021-07-10

**Authors:** Kum-Kang So, Jeesun Chun, Nguyen Ngoc Luong, Hee-Won Seo, Dae-Hyuk Kim

**Affiliations:** 1 Institute for Molecular Biology and Genetics, Department of Molecular Biology, Department of Bioactive Material Sciences, Jeonbuk National University, Jeollabuk-do, Jeonju, 54896 Republic of Korea; 2 Department of Biology, College of Sciences, Hue University, Hue, Vietnam

**Keywords:** Immunocomplex, *Saccharomyces cerevisiae*, Dengue, Vaccine, Envelope domain III

## Abstract

**Objectives:**

To explore *Saccharomyces cerevisiae* as an expression platform for dengue oral immune complex vaccine development.

**Results:**

Molecular engineering was applied to create a fusion gene construct (scEDIII-PIGS) consisting of a yeast codon optimized sequence encoding for a synthetic consensus dengue envelope domain III (scEDIII) followed by a modified IgG Fc domain (PIGS). Northern blot showed transcription of the target gene, with a temporal expression pattern similar to those from previous work. Western blot showed assembly of various immune complexes from monomer to hexamer. Partial purification of scEDIII-PIGS was also attempted to demonstrate the feasibility of yeast system for immune complex vaccine development. Approximately 1 mg of scEDIII-PIGS can be produced from 1 l culture.

**Conclusion:**

This work demonstrated for the first time that various immunocomplex structures of our target protein could be efficiently produced in *S. cerevisiae* for future application in developing oral and injectable vaccines against various pathogens.

**Supplementary Information:**

The online version contains supplementary material available at 10.1007/s10529-021-03161-7.

## Introduction

Dengue fever is a disease caused by mosquito-borne dengue virus and becomes a major global health crisis. Dengue viruses consist of four serotypes that are genetically distinct but highly similar (Katzelnick et al. 2015). While infection with one dengue serotype confers life-time protection against that serotype, the most severe forms of dengue fever, dengue hemorrhage fever and dengue shock syndromes, are the result of secondary infection by heterologous serotypes, a process referred to as “antibody-dependent enhancement”, (Balsitis et al. 2010). Although a number of dengue vaccines are being developed, the molecular and immunological reasons behind poor performance are still being studied.

Mucosal vaccines are vaccines that can be delivered nasally, orally, rectally or vaginally. Theoretically, mucosal vaccines are cheaper, safer, and more appropriate for mass distribution than parenteral vaccines (Lycke 2012; Zeng 2016; Miquel-Clopés et al. 2019).

Among various delivery systems for mucosal vaccines, *Saccharomyces cerevisiae* represents a very attractive choice. Yeast is a GRAS organism and long used as a food supplement. Moreover, *S. cerevisiae* is a cost-effective eukaryotic heterologous expression host. Finally, yeast cells are sufficiently immunogenic in human as a result of carbohydrate molecules on their cell wall that act as Pathogen Associated Molecular Patterns (PAMPs) (Kumar and Kumar 2019).

Immunocomplexes (ICs) are formed when antibodies like IgG or IgM bind to their cognate antigens. ICs may work as vaccines, show superior immunogenicity compared with alum-precipitated antigens, and also induce stronger cellular and humoral immune responses (Hioe et al. 2009; Kumar et al. 2013). Recombinant DNA technology allows for convenient production of recombinant ICs in which antigen fused Fc fragments are capable of forming more complex structures. These structures are collectively referred to as PIGS (Polymeric IgG Scaffolds).

Although several expression hosts, including plant and CHO cells, have been used to express PIGS-based antigens (Kim et al. 2017, 2018), no study has used yeast as an expression host for PIGS. In this study, we explored the possibility of using *S. cerevisiae* to produce an immunocomplex (scEDIII-PIGS) comprised of a synthetic consensus dengue envelope domain III (scEDIII) conjugated with a murine Fc fragment from IgG2 (PIGS). The scope of this study is limited to the expression and purification of the scEDIII-PIGS for subsequent use in the development of dengue vaccine.

## Materials and methods

### Strains and culture conditions

All plasmids used in this work were maintained in *Escherichia coli* Top10 strain. BL21 (de3) RIPL was used for the production of synthetic consensus Dengue envelope domain III antigen (scEDIII) in *E. coli*. The *E. coli* strains were maintained in Luria Bertani broth supplemented with appropriate antibiotics. The *Saccharomyces cerevisiae* 2805 strain (*MATα pep4::HIS3 prb 1-δ Can1 GAL2 his3 ura3-52*) used previously by our group in other yeast-based oral vaccine development work (Kim et al. 2011; Bal et al. 2018a, b) was used as the host for scEDIII-Fc complexes (scEDIII-PIGS). The recipient strain was maintained in YEPD liquid and agar media. The transformants were maintained in uracil dropout media (Ura^−^), which was comprised of yeast nitrogen base with ammonium sulfate (0.56% w/v), KCl (0.76% w/v), glucose (2% w/v), and yeast synthetic dropout (0.14% w/v), and supplemented with 20 mg of tryptophan, histidine, leucine, and adenine hemisulfate.

*Escherichia coli* and *Saccharomyces cerevisiae* chemical transformations were employed as previously described (Nguyen et al. 2013; Bal et al. 2018a). For scEDIII-PIGS production, a single colony of the transformant was inoculated into 5 ml of Ura^−^ liquid medium for 48 h in a shaking incubator at 200 rpm and 30 °C. 250 µl of this seed culture was transferred into 5 ml of YEPD medium and cultured for 16 h in identical conditions. This culture was subsequently inoculated into a baffled Erlenmeyer 300 ml flask containing 40 ml of YEPD medium and further cultured until cells were harvested for expression analysis.

### Construction of the expression vector

The expression construct encoding for the scEDIII-PIGS was created by overlap extension PCR between the gene encoding yeast codon-optimized scEDIII (GenBank ID: DI43185731, Nguyen et al. 2013) and the gene encoding murine-modified IgG2a-Fc fragment (Kim et al. 2017). The fusion construct was cloned into a pGEM-T Easy Vector system and its sequence confirmed. This fusion construct was subsequently cloned into the yeast episomal vector pYEGPD-TER (Lim et al. 2003) using *Bam*HI and *Sal*I restriction enzymes. The resulting recombinant vector (pYEGPD-scEDIII-PIGS) was sequenced with a primer pair flanking the expression construct and the result was compared with the reference sequence to ensure the accuracy of the ORF (Supplementary Fig. 1 and Supplementary Table 1).

### Colony PCR and back transformation

Colony PCR was used to confirm the presence of the pYEGPD-scEDIII-PIGS episomal plasmid. *Escherichia coli* back transformation was employed to ascertain the integrity of the expression cassette after yeast transformation as described previously (Nguyen et al. 2013).

### Expression analysis

The expression of scEDIII-PIGS was examined at the transcription and translation level. Northern blot analysis was conducted to detect the accumulation of the scEDIII-PIGS transcript. For the Northern blot analysis, 20 randomly selected transformants were cultured for three days and their total RNA was analyzed for the presence of the scEDIII-PIGS mRNA to determine the transformants with the highest transcription levels. The two transformants displaying the strongest hybridizing bands were selected and subsequently analyzed for their temporal expression pattern at the transcription and translation levels (1-, 3-, and 5-days post-inoculation).

### Northern blot analysis

Total yeast RNA preparation was carried out using the procedure described by Lim et al. (2003). RNA concentrations were determined using 96 microplates in the Multiskan GO UV spectrophotometer (Thermo Fisher Scientific Inc.) and approximately 30 µg of total RNA from each yeast transformant was analyzed on 1.2% (w/v) denatured formaldehyde-agarose gel. RNAs were blotted onto an Amersham Hybond™ nylon membrane (GE Healthcare) according to the manufacturer’s instructions. Hybridization was carried out in modified Church buffer (Nguyen et al. 2013) with a scEDIII-PIGS PCR product as a detecting probe.

### Protein preparation, SDS-PAGE and Western blot analysis

Approximately 500 µl of wet cell mass was mixed with 400 µl of 0.5-mm diameter acid washed glass bead (BioSpec Products Inc.) and 600 µl of breaking buffer (200 mM Tris–HCl pH 8.0, 150 mM ammonium sulfate, 1 mM EDTA and 10% v/v glycerol). The mixture was homogenized using FastPrep24™ 5G bead beater (MP Biomedicals) by beating for 30 s 8 times with a 1-min cooling interval between beatings. The lysate was first centrifuged at 7000 rpm for 20 min at 4 °C and the supernatant was collected and further clarified by 20-min centrifugation at 13,000 rpm. After concentrations were determined by Bradford assay, the protein samples were stored at − 20 °C for subsequent analysis.

For SDS-PAGE and Western blot analysis approximately 100 µg or 200 µg of total protein from each putative transformant was analyzed on a discontinuous SDS-PAGE gel consisting of 5% (v/v) stacking gel and 10% or 12% (v/v) separating gel. For samples in a denatured condition, samples were boiled for 10 min prior to the loading on the gel. For samples in a non-denatured condition, samples were loaded on the gel after being mixed with 6 × loading buffer without dithiothreitol (non-reducing 6 × loading buffer). The gels were subsequently blotted onto a 0.4 µm Nylon membrane and the target protein was detected by anti-dengue antibody (LSBio).

### Purification of the target proteins

Functional monomeric and polymeric scEDIII-PIGS were purified at a small scale using Pierce Protein A agarose (Thermo Fisher Scientific Inc.). Eluent fractions were collected in 1 ml each and subsequently concentrated with either 5 × (v/v) cold acetone or Amicon Ultra-15 centrifugal filter (Merck Millipore). Alternatively, the proteins were purified with Bioneer’s Maglisto magnetic protein A kit. The eluents were analyzed on 8% (v/v) SDS-PAGE gel in a non-denatured condition (without boiling in non-reducing 6 × loading buffer) and the gel was blotted on a Nylon membrane. Purified and active scEDIII-PIGS were detected as described above.

## Results

### Cloning and characterization of the expression cassette

**Fig. 1:**
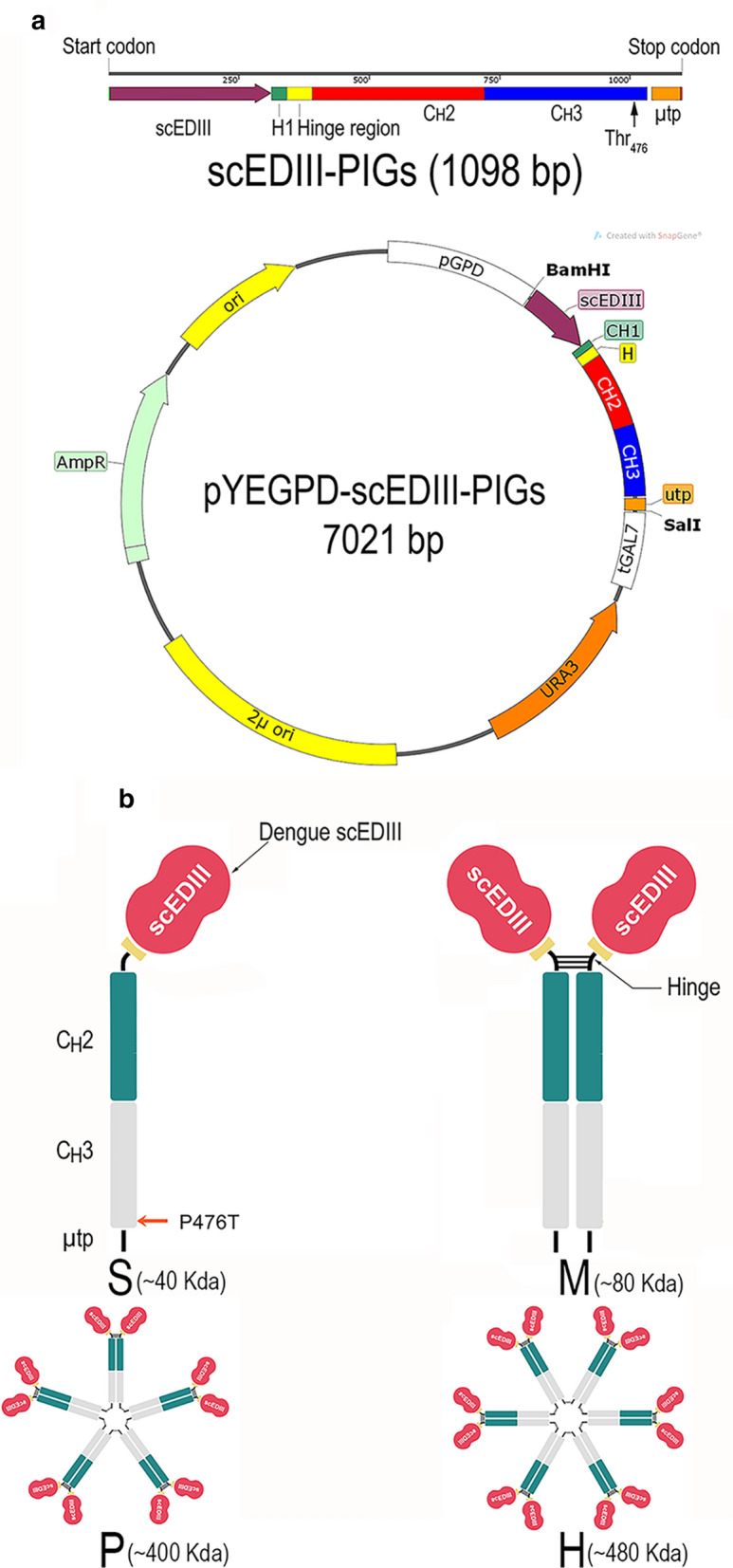
**a** Schematic representation of the genetic construct that encode scEDIII-PIGS and the recombinant plasmid pYEGDP-scEDIII-PIGS. **b** Schematic diagram of expressed scEDIII-PIGS from single chain (S) and monomeric (M) to polymeric [pentameric (P) and hexameric (H)] scEDIII-PIGS

In this study, the episomal vector yEPGPD-TER, which has already been used to express various antigens in yeast (Kim et al. 2011; Nguyen et al. 2013, 2015; Bal et al. 2018a, b), was employed to express the target gene. This vector is a high copy number vector containing a 2 µ yeast replication origin sequence that allows the vector to replicate independently from chromosomal DNA. The scEDIII-PIGS construct consists of a yeast codon-optimized gene that encodes a proof of concept tetravalent dengue antigen (Nguyen et al. 2013) fused with a sequence that encodes a mouse IgG2 Fc fragment. The Fc fragment contains extra amino acids at the N terminus, crossing the hinge region and the final ten amino acids of the C_H_1 domain, as well as an IgM µ tail piece (µtp) at the C-terminus. Furthermore, a Pro^476^ to Thr^476^ substitution (Pro476Thr) was performed in order to make the final three or four amino acids at the C-terminus of the IgG2 Fc fragment identical to those of the IgM Fc fragment (Kim et al. 2017). The scEDIII-PIGS construct is under the control of the strong constitutive glyceraldehyde-3-phosphate dehydrogenase (GPD) promoter and the galactose-1-phosphate uridylyltransferase (GAL7) terminator (Fig. 1).

### Expression and assembly of the target gene product

**Fig. 2:**
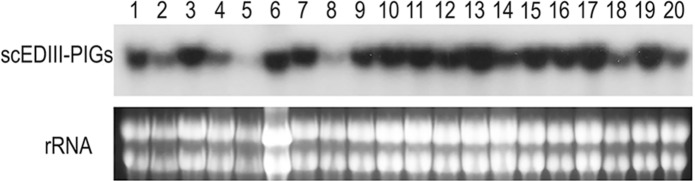
Northern blot analysis of scEDIII-PIGS transcript. 20 randomly selected transformants were analyzed. Above: Northern blot signals showing the transcription of scEDIII-PIGS expression cassette. Bellow: rRNA electrophoresis figure was shown to indicate equivalent amount of total RNA for each transformant was compared. Each lane contained 30 µg of total RNA. From this result, two strongly expressed strains (transformants #13 and #17) were selected for further analysis

**Fig. 3:**
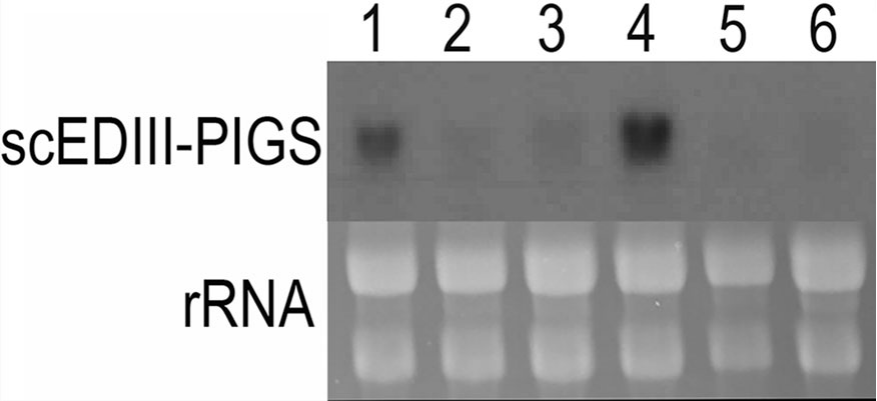
Temporal expression pattern of scEDIII-PIGS transcripts from two selected transformants #13 and #17. (Lanes 1, 2, and 3): total RNA from strain #13 at 1-, 3-, and 5-days post-inoculation; (Lanes 4, 5, and 6): total RNA from strain #17 at 1-, 3-, and 5-days post-inoculation. Each yeast sample analyzed in this experiment contained 30 µg of total RNA. rRNA electrophoresis bands are shown to indicate equal amount of total RNA

Transformed yeast cells were selected on the Ura^−^ medium. The transformation efficiency was extremely high, with 10 µl of competent cells yielding more than 100 putative transformants per plate. When the putative transformants were analyzed by colony PCR, all transformants showed an expected band at approximately 1 kb with the exception of the mock transformant (data not shown). *Escherichia coli* back transformation and subsequent sequence analysis of the pYEGPD-scEDIII-PIGS showed that the integrity of the expression cassette was preserved during the chemical transformation (data not shown). Northern blot analysis (Fig. 2) of 20 randomly selected putative transformants revealed successful transcription of the target gene in all examined transformants. Based on the Northern blot analysis, two strains with the strongest transcription levels were selected for their temporal expression pattern. The results showed that the scEDIII-PIGS transcription level was highest at day 1 and then decreased rapidly at day 3 post-inoculation (Fig. 3). This is consistent with our previous results (Nguyen et al. 2013; Bal et al. 2018a) and may be explained by the fact that in nonselective media, episomal plasmids will gradually dilute out.

**Fig. 4:**
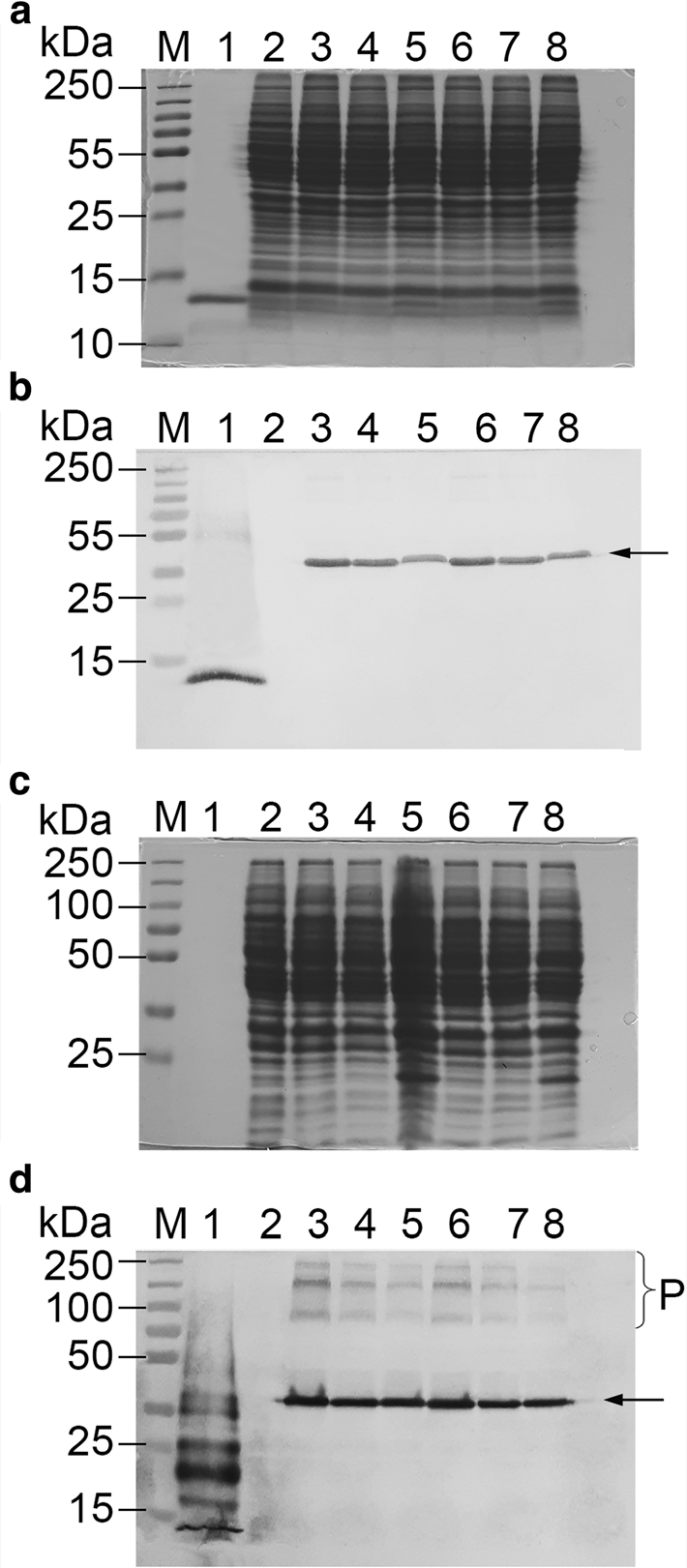
Temporal expression analysis by Western blot of two selected strains (transformants #13 and #17) under denatured (boiling) condition (**a** and **b**) and nondenatured (non-boiling) condition (**c** and **d**). SDS-PAGE images (**b** and **d**) are shown to indicate equal amount of protein for each sample. M: Pageruler prestained protein ladder (Thermo Fisher Scientific Inc.). **a** and **b** lane 1: 250 ng of purified *E. coli* expressed scEDIII; lanes 2, 3, and 4: total soluble protein from transformant #13 at 1-, 3-, and 5-days post-inoculation, respectively; lanes 5, 6, and 7: total soluble protein from transformant #17 at 1-, 3-, and 5-days post-inoculation, respectively; lane 8: total soluble protein from mock transformant. **c** and **d**: lane 1: total soluble protein from mock transformant; lanes 2, 3, and 4: total soluble protein from transformant #13 at 1-, 3-, and 5-days post-inoculation; lanes 5, 6 and 7: total soluble protein from transformant #17 at 1-, 3-, and 5-days post-inoculation; lane 8: 250 ng of purified *E. coli* expressed scEDIII. All yeast samples contain equal amount (100 µg in a and b and 200 µg in **c** and **d**) of total soluble protein. Arrows point to the positions of single chain scEDIII-PIGS (lower) while brackets (upper) indicate monomeric to polymeric (putatively pentameric/hexameric) forms on SDS-PAGE gels

Western blot analysis of the cell free extracts from the selected two strains with the strongest transcription at different time points (1-, 3-, and 5-day post-inoculation) displayed the distinctive antibody-reacting band at the expected size of 40 kDa under the denatured (sample-boiling) conditions, corresponding to the single chain of scEDIII-PIGS (Fig. 4a, b). Without question, the protein product of the target gene was successfully expressed. Although analysis of deduced amino acid sequences of scEDIII-PIGS did reveal the presence of two canonical N-glycosylation sites (Asn-X-Ser/Thr) at amino acid residues of 196–198 and 351–353, the lack of a significant difference in the size of the single chain of scEDIII-PIGS from the estimated size on the gel, in conjunction with the absence of microheterogeneity of expressed scEDIII-PIGS, suggested that glycosylation may not be strong in scEDIII-PIGS. Consistent with the Northern blot analysis, protein production (represented by the intensity of antibody-specific bands at 40 kDa under denatured condition) was strongest at day 1 and weaker by day 3 and day 5 (Supplementary Table 2). Based on the relative intensity of the antibody binding band in the Western blot, we estimated that 1 l of yeast culture yields approximately 1 mg of scEDIII-PIGS.

More importantly, in non-denatured condition, several additional bands that correspond to single chain scEDIII-PIGS (~ 40 kDa), monomeric scEDIII-PIGS (~ 80 kDa), and polymeric scEDIII-PIGS (~ 160 to 480 kDa) were observed (Fig. 4c, d). The band intensity of these polymeric scEDIII-PIGS forms was more distinct at day 1 than at days 3 and day 5, which also suggests increased expression and assembly at the early expression stage. Antibody-specific binding, and the appearance of single and multiple band patterns depending on the denatured- and non-denatured condition of sample proteins are distinct characteristics of PIGS-based expression, and have been previously described (Kim et al. 2017, 2018). This further confirms that yeast-expressed scEDIII-PIGS are expressed well and, moreover, are able to be assembled into polymeric structures when in a non-denatured condition.

### Purification of scEDIII-PIGS and evaluation of their potential for vaccine development

**Fig. 5:**
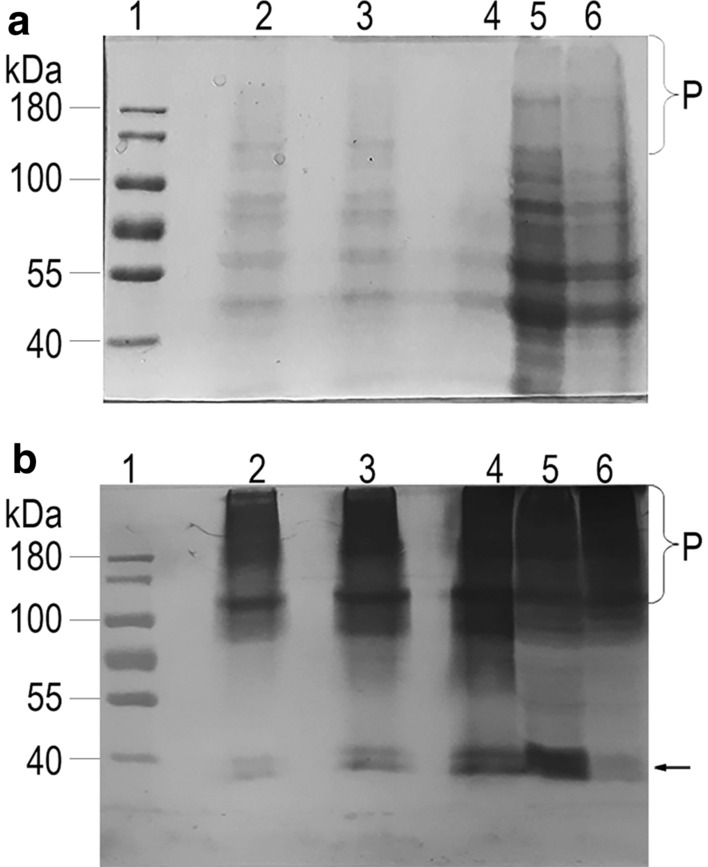
Western blot analysis of scEDIII-PIGS purification. **a** SDS-PAGE analysis of samples under non-denatured conditions; **b** Western blot analysis from the corresponding SDS-PAGE gel. Lane 1: protein ladder (Thermo Fisher Scientific Inc.); lane 2: Elution fraction 3; lane 3: Elution fraction 2; lane 4: elution fraction 1; lane 5: flowthrough; and lane 6: total protein. Brackets (upper) and an arrow (lower) indicate monomeric to polymeric (putatively pentameric/hexameric) forms and single chain scEDIII-PIGS, respectively, on SDS-PAGE gels

scEDIII-PIGS purification relies on the premise that scEDIII-PIGS behave like immunoglobulins due to their common Fc fragment. Indeed, we were able to partially purify scEDIII-PIGS to using either protein A conjugated magnetic beads or protein A conjugated agarose gravity flow columns. SDS-PAGE and Western blot analysis showed a distinct band at approximately 100 kDa, which probably correspond to the monomeric scEDIII-PIGS, and an intense smearing above that band but not below it, which corresponds to dimeric, pentameric, and hexameric forms (Fig. 5). This smearing pattern of high molecular weight forms of scEDIII-PIGS agreed with previous works expressing scEDIII-PIGS in tobacco and animal cells (Kim et al. 2017, 2018).

## Discussion

Monoclonal antibody (mAb) production has revolutionized biotechnology, with mAbs currently accounting for 40% of the global biopharmaceutical market (https://www.datamintelligence.com/research-report/biopharmaceuticals-market). While a great deal of research has focused on the antigen binding portion of antibodies, attention is increasingly being paid to the possibility of exploiting the biological functions of the Fc fragment (DiLillo et al. 2015; Blundell et al. 2017; Mackness et al. 2019; Kariolis et al. 2020). New classes of biopharmaceuticals include therapeutic Fc-fusion proteins, which improve half-life and efficiency. To date, thirteen such biopharmaceuticals have been approved in Europe and the United States (Duivelshof et al. 2020). Interest in the Fc fragment has also been focused on the potential use of the immunocomplex to vaccinate against various infectious diseases and contribute the fight against cancers (Hioe et al. 2009; Kumar et al. 2013; DiLillo et al. 2015; Kim et al. 2015; Kim et al. 2017).

Mammalian bodies contain five classes of immunoglobulin; IgM, IgG, IgA, IgE and IgD. The Fc fragment associated with each isotype distinguishes each from the others and confers its distinct biological functions. IgMs are the first isotype that are secreted by B cells (Kubagawa et al. 2019). They have low antigen affinity but high avidity due to their polymeric nature. IgMs can assemble into pentamers in the presence of J chain or hexamer in the absence of J chain (Brewer and Corley 1997). In-depth study of the principles of IgM hexamer formation showed that C_H_4 and the µtp are sufficient preconditions for its hexameric formation (Müller et al. 2013). Interest in IgMs grew once it was proposed that it might be possible to engineer Fc from IgG, and that this might result in a hybrid IgM-IgG Fc that allowed for multimeric assembly and possessed the desirable properties of both IgM and IgG (Smith and Morrison 1994). Previous studies created hexameric IgG by grafting a modified IgG C-terminal tail piece to the IgG C_H_3 domain. Ultimately, this process resulted in the successful production of multimeric sCEDIII-PIGS in *Nicotiana benthamiana* and CHO cells (Kim et al. 2017).

Vaccines are hailed as one of the most important discoveries of the previous century, and have saved millions of lives. Unfortunately, there remain many diseases where vaccines are either unavailable or ineffective, including HIV, malaria, and cancer. Dengue is currently the most serious mosquito borne infection (Diamond and Pierson 2015), and while some dengue vaccines have reached the clinical trial stage and even received regulatory approval, no vaccine has gained universal acceptance (Idris et al. 2020). With the ongoing SARS-CoV-2 pandemic taking up so many public health resources, the imperative of improving the vaccine development process to prevent other infectious diseases is very clear. An immunocomplex approach such as scEDIII-PIGS has significant potential as a novel vaccine development approach for a number of diseases, but particularly for the development of an oral Dengue vaccine.

Although expression using *S*. *cerevisiae* has the same intrinsic limits associated with all heterologous proteins, i.e., questioning the assembly authenticity of the expressed protein and posttranslational modification, it offers significant advantages to vaccine development over other expression systems, as yeast cells are suitable to be taken up by antigen-presenting cells (APCs) (Bernstein et al. 2008) and are potent transporters into dendritic cells, triggering antigen-specific CD4 + and CD8 + immune responses in vivo (Howland et al. 2008; Liu et al. 2016). In addition, *S*. *cerevisiae* tends to trigger potent innate as well as adaptive T cell immune responses (Bucarey et al. 2009; Bal et al. 2018a). In our previous papers, we used a yeast-expressed synthetic consensus sequence of EDIII (scEDIII) as a proof-of-concept Dengue tetravalent antigen to test various strategies for developing tetravalent Dengue vaccines. We showed that purified scEDIII from recombinant yeast induced a balanced immune response in mice and that when it was conjugated with gut mucosal layer binding ligands such as Co1 or LTB, it had immense potential as a recombinant tetravalent Dengue oral vaccine (Nguyen et al. 2013; Bal et al. 2018a, b). Building on our success of strong immunogenicity and neutralizing activity we previously achieved using yeast-expressed scEDIII, in this work we attempted to produce an immune-complex-like structure (scEDIII-PIGS) using *S. cerevisiae*. The simplicity of this system, which consists of an episomal vector (yEPGDP-TER) transformed into an auxotrophic mutant yeast strain (2085, ura3^−^) using LiAc method, means that various strategies for vaccine development can be attempted at very low cost and in a very time-efficient manner. Although it is clear from the Western results that immune complexes have formed successfully, the expression level can still be improved further using different combinations of promoters and terminators since we can see from the Northern blot results that the transcription level of the target gene dropped markedly over time. Expression level is a crucial factor since this synthetic complex is intended to work as a Dengue oral vaccine candidate. The appearance of the monomeric product at approximately 100 kDa may reflect the atypical movement of the protein in SDS-PAGE under non-reducing conditions instead of changes in molecular weight since the single chain scEDIII-PIGS moved with expected molecular weight. Indeed, under non-boiling but reducing conditions, we observed distinct bands at expected sizes (data not shown).

Our expression level of scEDIII-PIGS in yeast system appears to be comparable to the same protein expressed in *N. benthamiana* and CHO cells by Kim et al. (2017). While the same protein construct expressed in plants and CHO cells have been previously shown to work extremely well as a parenteral vaccine (Kim et al. 2017, 2018), it still required alum adjuvant. Apparently, injection is not desirable for dengue vaccines as resource limited countries simply lack the capacity to successfully implement an injection-based mass dengue vaccination program. An intrinsically adjuvanted, cheap, and convenient system such as *S cerevisiae*, however, which produces immune-complex at a high expression level and offers a various customizable genetic toolkit that permits further engineering, can form the basis of an effective oral vaccine.

## Conclusion

This study shows for the first time that various immunocomplex structures of our target protein, ranging from monomer to hexamer, can be efficiently produced in *S. cerevisiae*. We cloned a fusion gene construct encoding for murine scEDIII-PIGS using a high-copy yeast episomal expression vector, pYEGPD-TER, which we used to transform the *S. cerevisiae* 2805 strain. Successful expression was observed via Northern and Western blot analysis. Temporal expression analysis showed that scEDIII-PIGS expression peaked on day one and decreased over time, possibly due to the loss of the episomal vector under a non-selective condition. Notably, the Western results revealed the presence of polymeric scEDIII-PIGS, which can be purified using protein A affinity chromatography. The final yield of scEDIII-PIGS is estimated to be approximately 1 mg per liter of culture. scEDIII-PIGS, which are potent immune complex dengue vaccines, are produced in *S. cerevisiae* at a level that would potentially warrant mass adoption of this method and allow for the widespread distribution of this novel dengue vaccine. The resulting vaccine may be delivered orally as crude cell extract or parenterally as purified immune complexes.

## Supplementary Information

Below is the link to the electronic supplementary material.

Supplementary file1 (DOCX 683 kb)
