# Supplementary material for: Expression of an immunocomplex consisting of Fc fragment fused with a consensus dengue envelope domain III in Saccharomyces cerevisiae
Source: Biotechnol Lett. 2021 Jul 10;43(9):1895–904. doi: 10.1007/s10529-021-03161-7 (PMC8272446; doi:10.1007/s10529-021-03161-7)
Supplement: Supplementary file 1 — Supplementary file1 (DOCX 683 kb) [file 10529_2021_3161_MOESM1_ESM.docx]

**
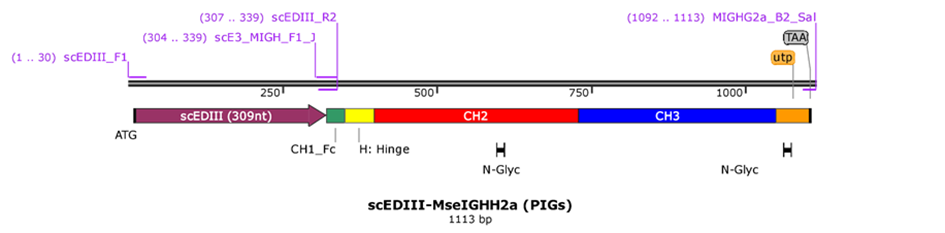
Supplementary Figure 1**. Positions of primers used in this study

| **Primer name** | **Primer sequences (5’ 🡪 3’)** |
| --- | --- |
| scEDIII-F1 | GAAGGATCCATGAAAGGAATGTCTTACGCA |
| scE3­­_MIGH_F1_J | TTTAAAAAGGGTTCCTCAGCTTCATCTACAAAAGTG |
| scEDIII_R2 | CACTTTTGTAGATGAAGCTGAGGAACCCTTTTT |
| MIGHG2a_B2_Sal | GTCGACTTAGTAGCAAGTGCCA |

**Supplementary Table 1**. Primer information

*Underlines indicate the restriction enzyme sites.

**Supplementary Table 2**. Band intensity in Western blots (as measured by ImageJ 2) of total protein (reducing condition) and polymeric protein (non-reducing condition)

| **Sample name** | **Reducing condition** | **Non-reducing condition** |
| --- | --- | --- |
| Positive control | 10,556 |  |
| Sample 1 Day 1 | 4,824 | 28,785 |
| Sample 1 Day 3 | 3,682 | 17,044 |
| Sample 1 Day 5 | 2,621 | 8,734 |
| Sample 2 Day 1 | 4,662 | 23,561 |
| Sample 2 Day 3 | 3,460 | 9,039 |
| Sample 2 Day 5 | 2,683 | 5,108 |

**Reference**:

Rueden CT, Schindelin J, Hiner MC, DeZonia BE, Walter AE, Arena ET, Eliceiri KW (2017) ImageJ2: ImageJ for the next generation of scientific image data. BMC Bioinformatics 529.
